# Nano-silica from white silica sand functionalized with PANI-SDS (SiO_2_/PANI-SDS) as an adsorbent for the elimination of methylene blue from aqueous media

**DOI:** 10.1038/s41598-023-45873-y

**Published:** 2023-10-31

**Authors:** Mohamed A. Salem, Ibrahim A. Salem, Wafaa M. El-Dahrawy, Marwa A. El-Ghobashy

**Affiliations:** Chemistry Department, Faculty of Science, Tanta University, Tanta, 31527 Egypt

**Keywords:** Environmental sciences, Chemistry, Nanoscience and technology

## Abstract

Natural resources including sand are one of the best approaches for treating dye-polluted wastewater. The SiO_2_/PANI-SDS nanocomposite was synthesized by self-assembly and intermolecular interaction. The physicochemical features of the SiO_2_/PANI-SDS nanocomposite were explored by FT-IR, XRD, SEM, TEM, EDX, and N_2_ adsorption–desorption techniques to be evaluated as an adsorbent for the MB. The surface area of the SiO_2_/PANI-SDS is 23.317 m^2^/g, the pore size is 0.036 cm^3^/g, and the pore radius is 1.91 nm. Batch kinetic studies at different initial adsorbate, adsorbent and NaCl concentrations, and temperatures showed excellent pseudo-second-order. Several isotherm models were applied to evaluate the MB adsorption on the SiO_2_/PANI-SDS nanocomposite. According to R^2^ values the isotherm models were fitted in the following order: Langmuir > Dubinin–Radushkevich (D–R) > Freundlich. The adsorption/desorption process showed good reusability of the SiO_2_/PANI-SDS nanocomposite.

## Introduction

Synthetic organic dye effluent wastewater discharged into the surrounding environment without treatment creates severe environmental risks. Dyes are essential in daily life since they are used in industrial applications such as papermaking, cosmetics, textiles, and medications. One of the most widely used dyes is the methylene blue (MB) which is utilized as a biochemical colorant in printing, leather, and other industries. Residual MB effluent can lead to serious environmental and health problems due to its high toxicity, chemical stability, difficult biodegradation, and possible carcinogenicity. Various methods have been developed to remove dyes from aqueous solutions. These include redox reactions, extraction, chemical precipitation, ion exchange, and advanced oxidation processes although these procedures are expensive and need additional chemicals. Therefore, a significant focus is currently concentrated on low-cost and effective adsorbents with a simple synthesis procedure. The adsorption process has been recognized as an attractive method for water treatment due to its economic feasibility and recyclability of the adsorbent^1^.

Polyaniline-based nanocomposites are popular in wastewater treatment owing to their redox characteristics, processability, and active amine/imine groups, which display high electrostatic interaction with contaminants such as heavy metals and dyes^2^. Ding et al.^3^ synthesized PANI@ammonium persulfate (PS) composite to remove the recycled Cr (VI) and MB from synthetic dyeing wastewater. Siadatnasab et al.^4^ successfully developed three unique nanohybrid composites denoted as CuS@PANI/PW12, CuS@PANI/PMo12, and CuS@PANI/SiW12. CuS@PANI/PW12 was more effective than the other and was able to adsorb 93% of MB (25 mg/L) in 45 min with a high adsorption capacity of 83.3 mg/g for the MB. Wanga et al.^5^ assessed the adsorption behavior of MB on polyaniline/TiO_2_ which revealed an adsorption capacity of 458.10 mg/g. Yan et al.^6^ synthesized the PANI hydrogel using phytic acid as a dopant and cross-linking agent. The phytic acid provided several anionic phosphate groups which act as efficient adsorption sites for the MB molecules. The adsorption capacity of PANI hydrogel for the MB was 71.2 mg/g. This value is greater than that of free PANI nanoparticles (6.1 mg/g), PANI nanotubes (9.2 mg/g), and PANI nanotube-based silica composites (10.3 mg/g). Thus, the synthesis of innovative polyaniline nanocomposites continues to spark a lot of curiosity^7^.

Natural resource-based silica has grabbed the interest of researchers because of its low cost, eco-friendliness, bioactivity, and availability. Silica nanoparticles are synthesized by various methods such as ball milling, hydrothermal, chemical vapor deposition, microemulsion, and sol–gel. In these methods, the reaction is controlled under either acid or alkaline conditions in which metal alkoxides such as Tetraethoxysilane (TEOS) or inorganic salts like sodium silicate are used as precursors^8^. Since the silica prepared from sand is superior compared to the chemically prepared silica, the researchers directed their interests to the natural silica-based adsorbents, functionalized mesoporous SiO_2_, and SiO_2_ nano/micro-particles for the treatment of water from contaminants^9^. El-Sawy et al.^10^ used the sol–gel precipitation method to successfully create silica nanoparticles from Egyptian silica sand as a substitute precursor for silica in the nanometre range. The silica nanoparticles were subsequently functionalized by the NH_2_–Cu complex and used the SiO_2_–NH_2_–Cu (II)@SiO_2_ as a catalyst for MB degradation. The findings revealed that 0.05 g of the nanocomposite could degrade 93.4% in 30 min. Ingrachen-Brahmi et al.^11^ explored the adsorptive elimination of MB from aqueous solutions using silica gel synthesized from Algerian siliceous by-products (sand) whose maximum removal efficiency was 80% in 180 min. Liu et al.^12^ used the coal gasification fine slag as a raw material for synthesizing a carbon–silica mesoporous (CSM) composite with a surface area of 500 m^2^/g and pore volume of 0.54 cm^3^/g. The maximum capacity for MB adsorption by the CSMs reached 182.48 mg/g, indicating a significant prospect in dye removal application.

In this paper, the silica nanoparticles were prepared from the raw silica sand and then incorporated into a modified polyaniline with sodium dodecyl sulfate surfactant (SDS) to form a novel SiO_2_/PANI–SDS nanocomposite. This nanocomposite was synthesized and applied for the first time to remove the MB from aqueous solution. The removal process was investigated under several influence parameters to evaluate its efficiency and reusability. The use of the SiO_2_/PANI–SDS nanocomposite as an adsorbent for the purification of water is the novel strategy used in this work.

## Experimental

### Chemicals

**Figure 1:**
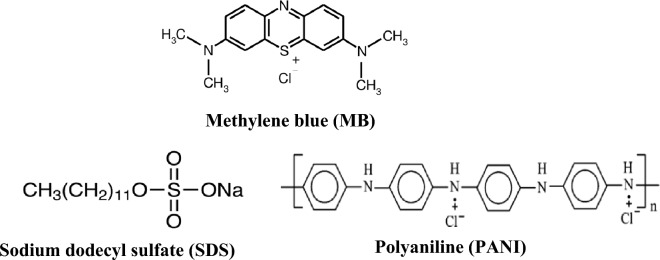
Structure of MB, SDS, and PANI.

The natural raw silica sand material was obtained from the Abu Zenima area, Sinai Peninsula, Egypt. The methylene blue (MB) (C_16_H_18_ClN_3_S) dye was obtained from Sigma Aldrich and used as received. Aniline (C_6_H_5_NH_2_, 99.5%) from Sigma Aldrich was distilled twice before being used. Ammonium peroxydisulfate (NH_4_)_2_S_2_O_8_ 98%) and sodium dodecyl sulfate, SDS, (CH_3_(CH_2_)_11_SO_4_Na 99%) are from Sigma Aldrich and used without further purification. C_2_H_5_OH 99%, HCl 37%, H_2_SO_4_ 99%, NaOH 98%, and NaCl 99.5% were of analytical grade reagents from Sigma Aldrich. The structures of MB and SDS are shown in Fig. 1. The distilled water was the main solvent over the entire work.

### Instrumental measurements

All materials were characterized by these instruments. FTIR spectra were recorded by JASCO FT-IR-4100 (Japan) spectrometer in the range of 4000–400 cm^−1^. The XRD was obtained by Brukeraxs D8, Germany. A copper Ka radiation target with a wavelength of 1.54 Å was employed. The morphology of the composite was examined using a scanning electron microscope (SEM: JEOL and JSM-6510LV) and a transmission electron microscope (TEM: JEM-2100 JEOL (Japan). The energy dispersive X-ray spectroscopy (EDX) was analyzed by an IT100LA operating at an accelerating voltage of 20.00 keV that was attached to the SEM device. The XPS analysis was made by XPS (PHI Quantera SXM; ULVAC-PHI, Inc., Osaka, Japan).

The specific surface area was measured using the BET (Belsorb III equipment, Japan) with $nitrogen$ as the adsorbate. The pH data were obtained with a Mettler Delta 320 pH meter. A UV/Vis double beam spectrophotometer (PG T80+, U.K.) was employed to monitor the progress of MB adsorption with time.

### Synthesis of PANI-SDS

The aniline (3 mL) was added to 10 mL of HCl (1 M) and stirred. Sodium dodecyl sulfate (SDS, 2 g) was dissolved in 50 mL of water. The SDS solution was added to the aniline solution and sonicated for 10 min. Ammonium peroxydisulfate solution (50 mL, 0.5 M) was added and magnetically stirred for 24 h to ensure complete polymerization of aniline. The obtained dark-green precipitate was filtered and rinsed several times with HCl (1 M), followed by water and alcohol before being dried in an oven at 60 °C for 12 h. These steps were repeated without using SDS to synthesize the pristine polyaniline (PANI), Fig. 1^13^.

### Synthesis of silica nanoparticles (SN)

The production of silica nanoparticles proceeds through two steps. The white sand is first purified, and then the pure silica is converted into nanoparticles^10^.

#### Natural white sand purification

Natural white sand (5 g) was washed several times with water to remove soluble contaminants. The sample was dispersed in HCl (5 M) under vigorous magnetic stirring at room temperature overnight. The sand was meticulously rinsed with sufficient water to eliminate the excess of HCl and the remaining free ions until pH 7. It was subsequently filtered and dried overnight in an oven at 100 °C.

#### Synthesis of silica nanoparticles (SN)

3 g of the pure silica and 5 g of NaOH pellets were crushed in a dry fusion procedure. The crushed material was fused for 4 h at 450 °C in a muffle furnace to form sodium silicate (Na_2_SiO_3_), which was leached with water according to Eq. (1). The solution was filtered to eliminate any unreacted silicate residue. This procedure was conducted twice to obtain a clear and colorless solution. H_2_SO_4_ (10 N) was then added to the Na_2_SiO_3_ solution under constant agitation to bring the solution to pH 9 and form SiO_2_ gel. Equation (2) indicates that the Na_2_SiO_3_ was hydrolyzed and condensed to form silica gel. The silica gel was rinsed with cold and hot water multiple times. It was dried at 80 °C for 32 h followed by 3 h at 120 °C. The solid material was crushed into powder using a plenary ball mill at 400 rpm for 3 h to hinder particle accumulation. It was then calcined in air at 400 °C for 1 h.

1 $${\text{SiO}}_{{\text{2}}} \left( {{\text{quartz sand}}} \right) + {\text{ NaOH}} \to {\text{Na}}_{{\text{2}}} {\text{SiO}}_{{\text{3}}},$$

2 $${\text{Na}}_{{2}} {\text{SiO}}_{{3}} + {\text{ H}}_{{2}} {\text{SO}}_{{4}} \to {\text{SiO}}_{{\text{2 gel}}} + {\text{Na}}_{{2}} {\text{SO}}_{{4}} + {\text{H}}_{{2}} {\text{O}}.$$

#### Synthesis of SiO_2_/PANI-SDS

**Figure 2:**
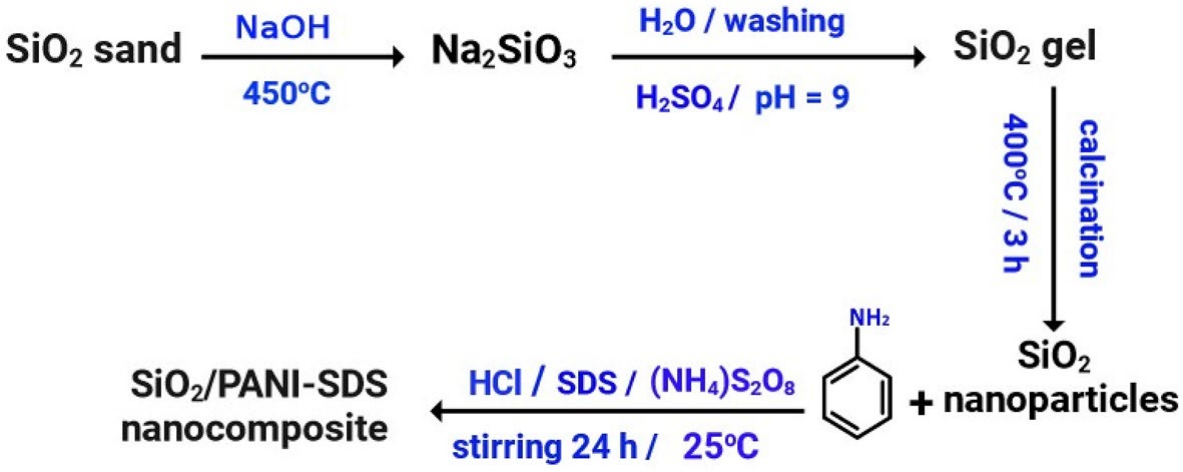
Synthesis of Silica nanoparticles and SiO_2_/PANI-SDS nanocomposite.

The SiO_2_/PANI–SDS nanocomposite was synthesized following the same procedure described in “Synthesis of PANI-SDS” section for synthesizing the PANI–SDS. Briefly, SiO_2_ nanoparticles (1.6 g) were added to the aniline-HCl solution, sonicated for 15 min, and magnetically stirred for 24 h. The black precipitate was rinsed several times with water and alcohol and dried overnight at 70 °C, Fig. 2.

### Procedure of MB adsorption

The MB adsorption run was conducted as follows: 1.06 mL of MB was withdrawn from its stock solution (300 mg/L) and transferred into an Erlenmeyer conical flask (100 mL), 48.8 mL of H_2_O, and 0.02 g of the SiO_2_/PANI–SDS nanocomposite were added, the total volume of the reaction mixture was 50 mL at pH 6. The flask was replaced into a shaking water thermostat at an agitating speed of 140 rpm and 30 °C. The time was noted at the moment when the nanocomposite was added to the reaction mixture. The kinetics measurements began immediately once the nanocomposite was added. Subsequently, 3 mL of the reaction mixture were withdrawn by a 0.45-μm Millipore filter to remove suspended particles, and the supernatant was transferred to a 1-cm quartz cell. The absorbance of the remained MB was then taken at λ_max_ = 665 nm. The tests were repeated until no additional change in the absorbance was detected. Finally, the removal efficiency was evaluated using Eq. (3), the amount of MB adsorbed at equilibrium was calculated by Eq. (5).

3 $$\mathrm{Removal \,efficiency \% }= \frac{{\mathrm{C}}_{o}-{\mathrm{C}}_{\mathrm{t}}}{{\mathrm{C}}_{o}}\times 100,$$

4 $${\text{q}}_{\text{t}}=\frac{{\mathrm{C}}_{o}-{\mathrm{C}}_{\mathrm{t}}}{{\mathrm{C}}_{o}} \times \frac{\mathrm{V}}{\mathrm{m}} ,$$

5 $${\text{q}}_{\text{e}}= \frac{{\mathrm{C}}_{o}-{\mathrm{C}}_{\mathrm{e}}}{{\mathrm{C}}_{o}} \times \frac{\mathrm{V}}{\mathrm{m}},$$

where C_o_ is the initial concentration of MB, C_e_ is the equilibrium concentration, and C_t_ indicates its concentration at time t. The q_t_ (mg/g) and q_e_ (mg/g) refer to the adsorption capacities of the SiO_2_/PANI-SDS nanocomposite at time t (min) and equilibrium.

### Reusability test

The previously used SiO_2_/PANI–SDS/MB nanocomposite was collected and separated from the medium by filtration. To explore the reusability of the SiO_2_/PANI–SDS nanocomposite toward the MB, the nanocomposite was suspended in 100 ml of ethanol–water mixture (30:70, mL) and stirred in a closed Erlenmeyer flask at 140 rpm for 12 h at 25 °C. Subsequently, it was centrifuged at 5000 rpm for 20 min, followed by repeated washing with water until a clear supernatant solution was obtained. Finally, the recycled SiO_2_/PANI–SDS nanocomposite was used in fresh adsorption runs under identical conditions. This procedure was repeated for six cycles.

## Results and discussion

### Characterization of adsorbent

#### FT-IR

**Figure 3:**
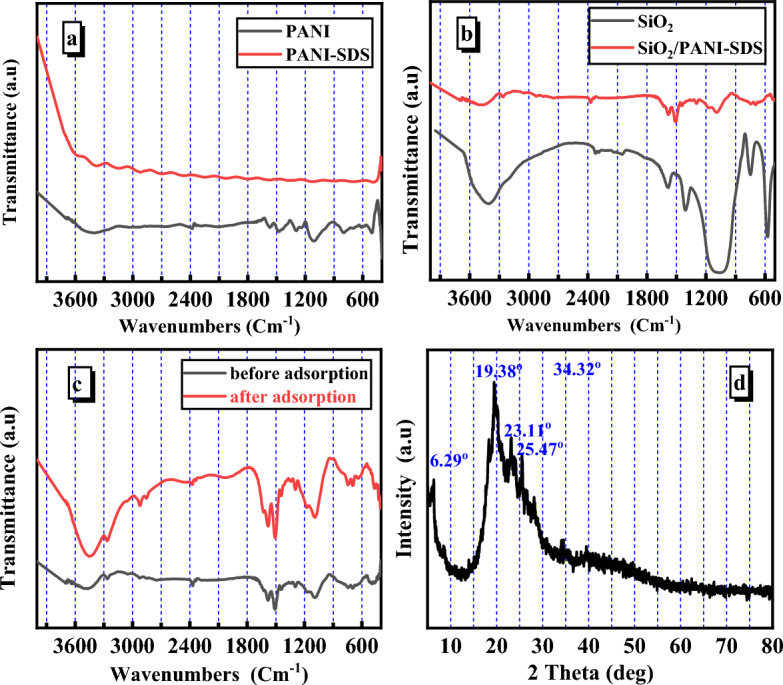
(**a**) FT-IR spectra of PANI, and PANI-SDS. (**b**) FT-IR spectra of SiO_2_, and SiO_2_/PANI-SDS nanocomposite, (**c**) FT-IR spectra of SiO_2_/PANI-SDS nanocomposite before and after MB adsorption. (**d**) XRD of SiO_2_/PANI-SDS nanocomposite.

Figure 3a,b depict the FT-IR spectra of PANI, PANI–SDS, SiO_2_, and SiO_2_/PANI–SDS nanocomposite, and the main unique bands of the four materials were assigned. The materials were explored in the range of 4000–400 cm^−1^. For the pure PANI, the peaks at 3400, and 1290 cm^−1^ are attributed to the secondary aromatic amine’s N–H and C–N stretching^14^. Meanwhile, the bands at 1470 and 1570 cm^−1^ are ascribed to the benzenoid and quinoid units^15^. The peak at 1108 cm^−1^ is attributed to the benzenoid ring’s C–N stretching mode, and the peak at 795 cm^−1^ is designated to the plane bending vibration of C–H, which is formed during the protonation^16^. The bands at 612 and 504 cm^−1^ can be assigned to the aromatic C–H out of the plane bending vibrations^17^. The PANI–SDS composite was subjected to FTIR analysis to confirm the successful functionalization of PANI with the SDS. In the FTIR spectrum of the PANI–SDS, the outstanding absorption bands at 3375 and 2922 cm^−1^ are ascribed to the C–H stretching vibrations of the SDS and N–H of the PANI^18^. The bands at 2925 and 2024 cm^−1^ confirmed the presence of the C–H stretching mode on the long alkyl tail of SDS and S=O extending of the SO_3_ group in the PANI–SDS. The peak at 1121 cm^−1^ is assigned to the vibration mode of the NH^+^ structure, showing the high degree of electron delocalization in the PANI–SDS and the strong interchain NH^+^–N hydrogen bonding^19^. The results indicate the successful formation of the PANI–SDS. The inclusion of SiO_2_ nanoparticles and SDS into the PANI chains induced the shifting of some PANI–SDS nanocomposites. The SiO_2_/PANI–SDS nanocomposite spectrum was compared to those of the PANI, PANI–SDS, and SiO_2_ to demonstrate its formation. The distinctive absorption of SiO_2_/PANI–SDS expresses a peak at 1585 cm^−1^ which is characteristic of the C=N in quinoid units and a peak at 3474 cm^−1^ for the N–H stretching. The peak at 3261 cm^−1^ belongs to the N–H stretching frequency of amine. The peaks at 495, 735, and 696 cm^−1^ are assigned to the Si–O bond, out of plane C–H bending, and out of plane C–C deformation vibrations in monosubstituted aromatic rings, respectively^20,21^. The strong peak at 1095 cm^−1^ describes the skeletal vibrations of the Si–O–Si obtained from silica^22^. The small peak at 2371 cm^−1^ indicates that carbon dioxide is adsorbed in the SiO_2_/PANI–SDS nanocomposite^23^. In summary, the discrepancies in the FTIR spectra of the PANI, SiO_2_, PANI–SDS, and SiO_2_/PANI–SDS nanocomposite guarantee the successful formation of the novel SiO_2_/PANI–SDS nanocomposite.

The FT-IR spectra of the SiO_2_/PANI-SDS nanocomposite before and after MB adsorption are compared in Fig. 3c to obtain additional information on the interaction between the SiO_2_/PANI–SDS nanocomposite and MB molecules. Although some of the FT-IR data are almost identical, others are relatively strengthened and displaced. The identical peaks at 1581, 1092, 1093, and 1296 cm^−1^ reveal the in-degradability of the SiO_2_/PANI–SDS nanocomposite after MB adsorption^24^. The peak at 3474 cm^−1^ is shifted to 3445 cm^−1^. It may be attributed to the stretching vibrations of the OH group. The novel peaks at 2922, 2856, and 2030 cm^−1^ correspond to the C–H asymmetric and symmetric stretching vibrations of the methylene blue molecules on the PANI/SDS/SiO_2_ nanocomposite surface, including the C–H of the aromatic ring^25^.

#### XRD

X-ray diffraction is a non-destructive method that assesses the phase purity and crystallinity of inorganic materials. Figure 3d depicts the diffraction peaks in the 2θ range 10°–70° and shows the amorphous structure of the PANI/SDS/SiO_2_ nanocomposite. The two peaks at 2θ = 19.38° and 25.47° correspond to the growth directions of (010) and (200) planes based on the JCPDS card no. 53-1891. They are linked to similar periodicity of the PANI polymer chain^26^. The peaks at 2θ = 17°, 23.11°, and 28.13° refer to the PANI-emeraldine salt state^27^. The XRD pattern of the nanocomposite indicates that the peaks are highly intense and count for the high crystallinity in the backbone of the PANI polymer^28^.

#### BET

**Figure 4:**
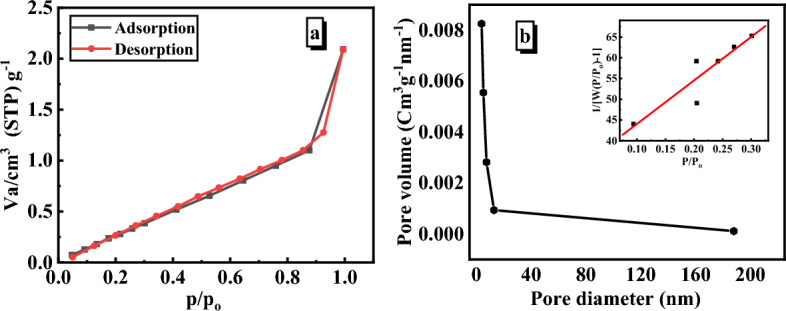
(**a**) N_2_ adsorption–desorption isotherms at 77.35 K. (**b**) BJH Pore size distribution and the insert is the multi-point plot of SiO_2_/PANI-SDS nanocomposite.

The surface area of the adsorbent material is critical to the extent of the adsorbent. It is therefore a quality characteristic. Following the IUPAC classification, the SiO_2_/PANI–SDS nanocomposite exhibits a type-IV isotherm with the usual H3 hysteresis loop of mesoporous materials, (Fig. 4a). The pore size distribution was also measured using the Barret, Joyner, and Halenda (BJH) method and is shown in Fig. 4b. The SiO_2_/PANI–SDS nanocomposite has a particular surface area of 23.317 m^2^/g, a pore volume of 0.035 cm^3^/g, and a pore radius of 1.91 nm^29^.

#### SEM, TEM, and EDX

**Figure 5:**
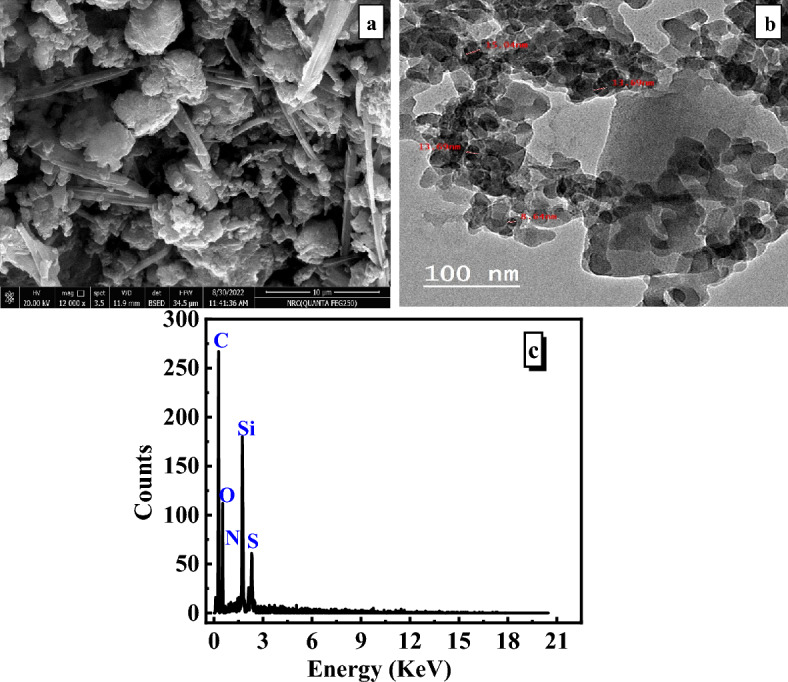
(**a**) SEM, (**b**) TEM, (**c**) EDX micrographs of SiO_2_/PANI-SDS nanocomposite.

SEM, TEM, and EDX analyses were performed to get more precise information on the particle morphology of the SiO_2_/PANI–SDS nanocomposite. The SEM image of the nanocomposite shows an asymmetrical microscopic scale with granular floccules, Fig. 5a. The TEM micrographs of the nanocomposite reveal a globular cluster of PANI matrix with SiO_2_ particles that have particle size in the range from 8 to 15 nm as given in Fig. 5b. The EDX analysis indicates the existence of silicon (6.28%) and oxygen (26.28%) from SiO_2_, carbon (54.31%), sulfur (3.29%), and nitrogen (9.21%) from PANI/SDS in the nanocomposite, Fig. 5c. The results show that the SiO_2_/PANI–SDS nanocomposite is effectively constructed from the five main components, namely S, Si, C, O, and N^30^.

#### XPS

**Figure 6:**
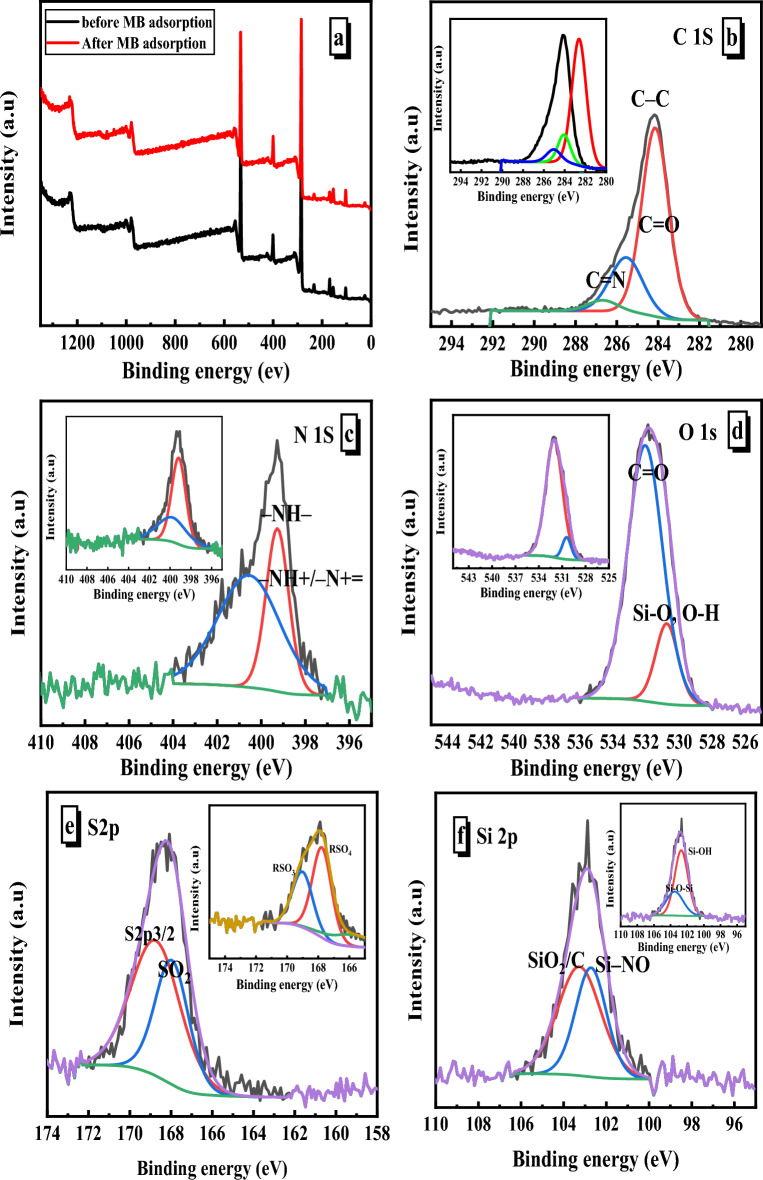
XPS spectra of SiO_2_/PANI-SDS nanocomposite: (**a**) full scan before and after MB adsorption, (**b**) C 1s, (**c**) N 1s, (**d**) O 1s, (**e**) S 2p, and (**f**) Si 2p. The insets are the spectra after adsorption.

The chemical nature and the composition of the SiO_2_/PANI-SDS nanocomposite were evaluated by X-ray photoemission spectroscopy (XPS). The XPS survey scan given in Fig. 6 shows the existence of five major peaks for C 1s peak at 285.38 eV, and O 1s peak at 532.81. eV, N 1s peak at 400.79 eV, S peak 2p, and Si 2p at 103,97 indicating the formation of SiO_2_/PANI–SDS nanocomposite. The atomic ratio of carbon, oxygen, nitrogen, sulfur, and silicon is 61.83:21.58:8.59:3.36:4.64. High resolution of XPS for C 1s ranging from 280 to 298 eV exhibits three peaks by deconvolution fitting of the C 1s spectrum. The major peak at 284.14 eV is responsible for the sp2 carbon atoms. The other two weak peaks at 285.54, and 286.67 eV correspond to C–S of SDS and C–N. The O 1s spectrum could be deconvoluted into two subpeaks at 530.75, and 532.6 eV, which could be attributed to the presence of different oxygen functionalities such as Si–O from silica nanoparticles, and S–O from SDS, respectively. The N 1s peak can be deconvoluted into two individual peaks at 399.27 eV, 400.55 eV assigned to N–H, and C–N of PANI, respectively. The S2p spectrum could be deconvoluted into two subpeaks at 168.2 eV and 168.47 eV which could be attributed to the presence of different sulfur functionalities such as S–O, and S–C from SDS, respectively. The Si 2p spectrum could be deconvoluted into two subpeaks at 102.95 eV and 103 eV which could be attributed to the presence of different Si environments such as Si–O, and Si–Si from silica nanoparticles, respectively. After the adsorption of MB into SiO_2_/PANI–SDS nanocomposite, the XPS survey shows that the composition has a little changed ratio of carbon, oxygen, nitrogen, sulfur, and silicon into 63.39:20.76:7.21:2.06:6.58 confirming the adsorption of MB on the surface of the SiO_2_/PANI–SDS nanocomposite. The peak deconvolution of each element is still the same indicating no changes in the functional groups of the nanocomposite.

### Adsorption study

#### Effects of adsorbent type

**Figure 7:**
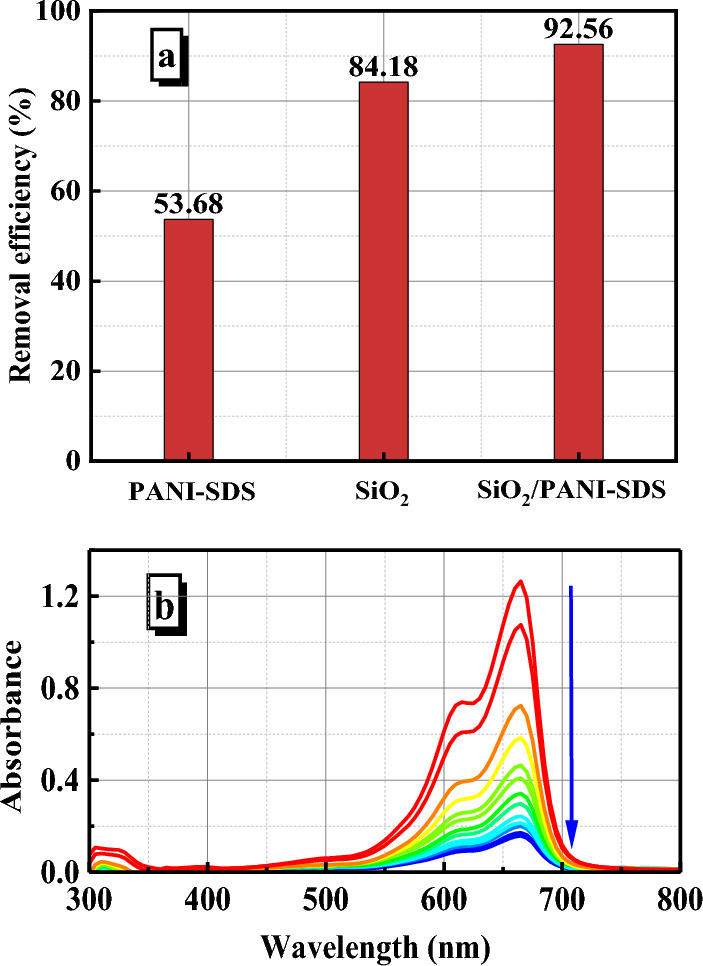
(**a**) Removal efficiency of MB by PANI-SDS, SiO_2_, and SiO_2_/PANI-SDS nanocomposite as adsorbents. (**b**) The decrease in absorbance of MB during adsorption on SiO_2_/PANI-SDS. [MB]_o_ = 6.36 mg/L, dose = 0.02 g, t = 30 min, pH 6 at 30 °C.

The present adsorption study determines which is the most active adsorbent among the SiO_2_, PANI–SDS, and SiO_2_/PANI–SDS nanocomposite for the removal of MB from aqueous solution. The removal efficiencies of these adsorbents were investigated under the same conditions, which were [MB]_o_ = 6.36 mg/L, dose = 0.02 g, pH 6, T = 30 °C, and stirring speed 140 rpm, Fig. 7a. The results revealed that the removal efficiency of PANI–SDS, SiO_2_, and SiO_2_/PANI–SDS adsorbents are respectively 53.68, 84.18, 92.56% and their adsorption capacities (q_e_) are 8.5, 13.39, and 14.97 mg/g, respectively. According to these results, it is clear that the SiO_2_/PANI-SDS nanocomposite is the most active adsorbent in the MB removal process, Fig. 7b. Hence, it was selected for the rest of the adsorption investigations.

#### Effect of dose

**Figure 8:**
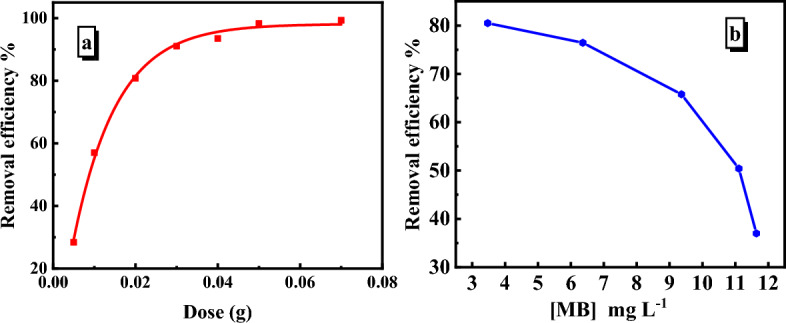
(**a**) Dependence of removal efficiency on the SiO_2_/PANI-SDS dose for the removal of MB at [MB]_o_ = 6.36 mg/L, (**b**) removal efficiency as a function of initial concentration of MB with adsorbent 0.02 g. Conditions: t = 14 min, pH 6, T = 30 °C.

The quantity of nanocomposite in the system significantly impacts the adsorption rate and is critical for preventing the waste material from desorbing after reaching equilibrium. The effect of nanocomposite dose was evaluated in the range from 0.005 to 0.04 g at a constant concentration of MB (6.36 mg/L), pH 6, and 30 °C with a constant stirring speed of 140 rpm. The results revealed that the SiO_2_/PANI–SDS nanocomposite can remove MB at a low dosage implying that it has adequate active sites for MB adsorption even if present in a small amount. Furthermore, the results showed that on increasing the nanocomposite dose from 0.005 to 0.04 g, the removal efficiency of MB raised from 28.39 to 93.52% within 14 min, Fig. 8a. This can be attributed to the increasing surface area of the nanocomposite and consequently the increasing number of adsorption sites that are available for MB adsorption. Such an increase in the removal efficiency of MB with the increasing dose continues rapidly until all the MB molecules get adsorbed on the nanocomposite surface. At this stage, the removal efficiency reaches an almost constant value at 0.04 g, indicating no more adsorption takes place^31^.

#### Effect of initial dye concentration

The effect of initial MB concentration was investigated at a constant dose of SiO_2_/PANI–SDS nanocomposite (0.02 g) and varied concentrations of MB in the range of 3.47–11.64 mg/L. As demonstrated in Fig. 8b, the MB removal efficiency dropped from 80.50 to 36.99% when the MB concentration was increased from 3.47 to 11.64 mg/L. Figure 8b reveals a rapid adsorption rate of the MB on the SiO_2_/PANI–SDS surface during the early stage and drastically reduced at the highest concentration of MB (11.64 mg/L). At low MB concentrations, the SiO_2_/PANI–SDS nanocomposite possesses accessible adsorption sites more than the present number of MB molecules. Therefore, the uptake of the molecules is fast and then the removal efficiency is high. Further, with the gradual increase in the MB concentration, the adsorption sites get occupied more and more with the MB molecules until the surface reaches the saturation state. In this case, the adsorption process of MB on the SiO_2_/PANI–SDS nanocomposite declines and hence the removal efficiency is dropped.

#### Effect of pH

**Figure 9:**
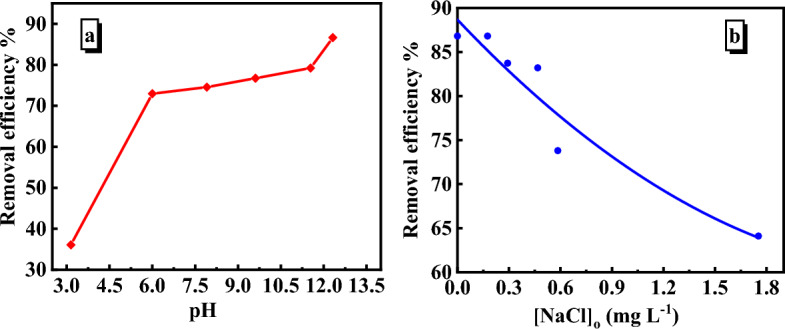
(**a**) The removal efficiency of MB at variable pH. (**b**) Effect of NaCl concentration on the removal efficiency of MB by SiO_2_/PANI-SDS. [MB]_o_ = 6.36 mg/L, pH 6, SiO_2_/PANI-SDS = 0.02 g, T = 30 °C.

The pH of the solution substantially impacts the adsorption process because it regulates the adsorbent surface charge, the magnitude of dye ionization, and the dissociation of functional groups on the adsorbent. The pH was varied from 3 to 12 to assess the effect of pH on MB adsorption. As seen in Fig. 9a, the increase in solution pH enhances the MB removal efficiency. The removal efficiency was maximum at pH 12 and minimum at pH 3. The law efficiency at pH 3 may be elucidated in two aspects. The first may be due to the protonation of the SiO_2_/PANI–SDS surface by the H^+^ ions present in solution, which enhance the electrostatic repulsion between the surface and the cationic MB molecules. The second may be linked to the competition between the H^+^ present in the medium and the positively charged MB molecules for the active adsorption sites of the nanocomposite. On increasing the pH, the removal efficiency increases owing to the increase in the negative charge density created on the surface, which in turn results in an electrostatic attraction between the SiO_2_/PANI–SDS surface and the positively charged MB molecules. Given these results, it seems that the electrostatic interaction taking place between the SiO_2_/PANI–SDS surface and the MB molecules over the entire investigated range of pH (3–12) is playing a predominant role in the adsorption process^32^.

#### Effect of salt

The effectiveness of the adsorption sites, the solubility, and the hydrophobicity of the dye are all impacted by co-existing salt, making it a vital component to investigate in the adsorption tests. The effect of NaCl on MB adsorption was evaluated at various NaCl concentrations ranging from 0.175 to 1.75 mg/L. When the NaCl concentration was increased from 0.175 to 1.75 mg/L, the MB removal efficiency dropped from 81 to 66% within 14 min, Fig. 9b. The reduction in the electrostatic contact between the MB molecules and the SiO_2_/PANI–SDS surface may be the cause behind the drop in removal efficiency. Furthermore, the Na^+^ ions and the cationic MB molecules compete for the active sites of SiO_2_/PANI–SDS, and the Na^+^ might comfortably occupy the active sites of the adsorbent faster than the MB molecules. This may be due to the smaller ionic radius of Na^+^ than that of the MB. Therefore, some of the MB molecules cannot reach the SiO_2_/PANI–SDS surface and then the removal efficiency is decreased^33^.

#### Effect of temperature

**Figure 10:**
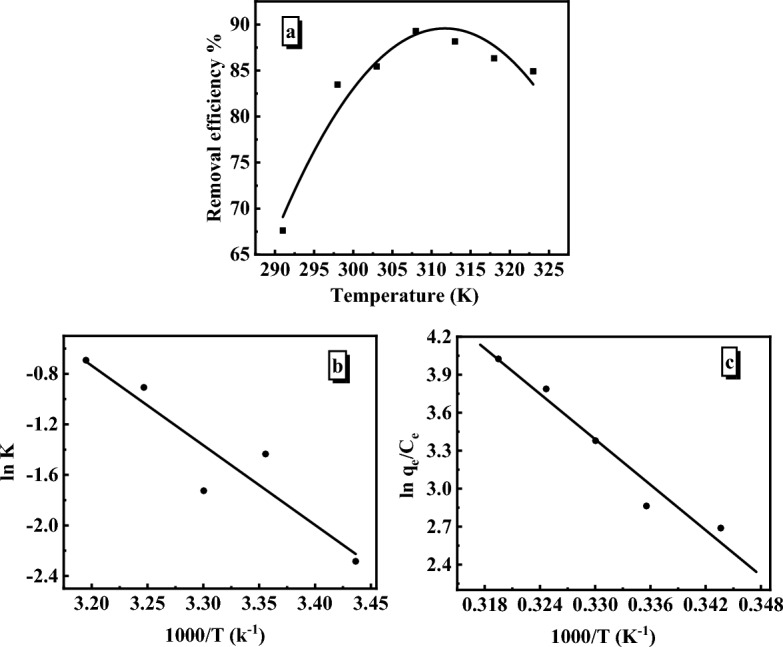
(**a**) Effect of temperature on the removal efficiency of MB by SiO_2_/PANI-SDS. [MB]_o_ = 6.36 mg/L, pH 6, SiO_2_/PANI-SDS = 0.02 g over 14 min. (**b**) Arrhenius plot. (**c**) Vant’s Hoff plot.

The influence of temperature on MB adsorption was investigated since it is one of the most significant elements affecting the adsorption rate. The effect of temperature was investigated at variable temperatures ranging from 291 to 323 K and fixed [MB]_o_ at 6.36 mg/L, pH at 6 rpm at 140, and SiO_2_/PANI–SDS dosage at 0.02 g. The results showed that as the temperature was elevated from 291 to 308 K, the MB adsorption increased from 67 to 89%, then declined slightly, Fig. 10a. The viscosity of the MB solution was lowered as the temperature was raised allowing the MB molecules to flow readily and migrate faster toward the nanocomposite surface. Therefore, the removal efficiency is increased. Furthermore, the slight decrease in the MB removal efficiency beyond 313 K may be due to the reduction of the attraction forces between the MB molecules and the active sites on the nanocomposite surface, resulting in a loss in the adsorption capacity^34^. As the temperature was raised, the volume of dissolved gas in the reaction mixture expanded, creating gaps between the MB molecules and the active sites of the SiO_2_/PANI–SDS. These gaps obstructed the MB movement to the surface of the SiO_2_/PANI-SDS nanocomposite. However, some of the MB molecules could not reach the nanocomposite surface and consequently, the removal efficiency is decreased^35^.

#### Activation parameters

The rate of most chemical reactions increases as the temperature increases. Therefore, the Arrhenius equation determines the activation energy (E) and the frequency factor (A) of the adsorption process. The Arrhenius equation is

6 $$\mathrm{ln\, k }=\mathrm{ ln \,A }- \frac{\mathrm{E}}{\mathrm{RT}},$$

where k is the pseudo-second-order rate constant (g mg^−1^ min^−1^), A is the Arrhenius factor, E is the activation energy, R is the ideal gas constant (8.314 J mol^−1^ K^−1^), and T (K) is the absolute temperature of the adsorption medium. The E and A values were obtained from the slope of ln k vs 1/T, Fig. 10b. The value of activation energy (E) shows whether the adsorption process of MB on the SiO_2_/PANI–SDS surface is physical or chemical. Physical adsorption is indicated by low activation energy (5–50 kJ/mol), whereas chemical adsorption is marked by high activation energy (60–400 kJ/mol). Since the current activation energy (51.86 kJ/mol) falls within the range of physical adsorption, then the MB adsorption on SiO_2_/PANI–SDS nanocomposite is a physical process. The enthalpy change (ΔH^⋕^ = 49.35 kJ/mol) indicates that the MB adsorption onto SiO_2_/PANI–SDS is endothermic. Furthermore, the low value of ΔG^⋕^ (69.86 kJ/mol) demonstrates spontaneous adsorption. The negative value of ΔS^⋕^ (− 230.58 J/mol) reveals less randomness in the adsorbed molecules of MB on the surface of the nanocomposite, Table 1^36^.

**Table 1:** Activation parameters of MB adsorption on SiO_2_/PANI-SDS, [MB]_o_ = 6.36 mg/L, dose = 0.02 g, pH 6 ± 0.1.

Temp (K)	k (L mol^−1^ min^−1^)	E (kJ mol^−1^)	ΔH^⋕^ (kJ mol^−1^)	ΔG^⋕^ (kJ mol^−1^)	ΔS^⋕^ (J mol^−1^ K^−1^)
291	0.101				
298	0.178				
303	0.238	51.86	49.35	69.86	− 230.58
308	0.403				
313	0.501				

#### Thermodynamic parameters

Thermodynamic parameters such as free energy change (∆G°) enthalpy, (∆H°), and entropy change (∆S°) provide detailed information regarding the energy changes that occur during the MB adsorption process. The following equations can be used to obtain these parameters.

7 $$\mathrm{ln\, K }= \frac{\Delta {{\mathrm{S}}^{\mathrm{o}}}_{ }}{\mathrm{R}}-\frac{\Delta {{\mathrm{H}}^{\mathrm{o}}}_{ }}{\mathrm{RT}},$$

8 $$\Delta {\text{G}}^{ \circ } \, = \, \Delta {\text{H}}^{ \circ } {-}{\text{ T}}\Delta {\text{S}}^{ \circ } ,$$

where, K (mg/g) is the standard thermodynamic equilibrium constant, T (K) is the absolute temperature, and R (J/mol K) is the gas constant. By graphing the linear plot between $\mathrm{ln\,K}$ vs. 1/T, the ∆S° and ∆H° values can be estimated, Fig. 10c. The thermodynamic parameters are calculated and presented in Table 2. The negative value of Gibbs free energy ∆G° (− 23.08 kJ/mol) suggests that MB adsorption occurs spontaneously and feasibly. The positive value of ∆S° (192.35 J/mol K) shows the improved randomness at the interface of the SiO_2_/PANI–SDS nanocomposite and the MB solution during the adsorption process. In contrast, the positive value of ∆H° (49.75 kJ/mol) suggests that the MB adsorption process was endothermic^37^.

**Table 2:** Thermodynamic parameters of MB adsorption on SiO_2_/PANI-SDS.

Temp (K)	ΔH° (kJ mol^−1^)	ΔG° (kJ mol^−1^)	ΔS° (J mol^−1^ K^−1^)
291			
298			
303	49.75	− 23.08	192.35
308			
313			

[MB]_o_ = 6.36 mg/L, dose = 0.02 g, pH 6.

### Adsorption kinetics

Important details about the adsorption mechanism can be revealed from the adsorption kinetics. Non-linear kinetic models like pseudo-first-order and pseudo-second-order were applied to analyze the MB adsorption kinetics on the SiO_2_/PANI–SDS nanocomposite. Based on an error study that include calculating the non-linear chi-square (χ^2^) and the Root Mean Square Error (RMSE), the best kinetic model was selected. The best model has an R^2^ value that is close to 1, a χ^2^ value that is close to 0, and a lower RMSE. The estimated coefficients are displayed in Table 3^39^.

**Table 3:** Pseudo-first-order and pseudo-second-order kinetic models for adsorption of MB on SiO_2_/PANI-SDS (0.02 g), pH 6 ± 0.1, T = 30 °C.

[MB]_o_ (mg/L)	q_e_._exp_ (mg/g)	Pseudo-first-order model				q_e_._exp_ (mg/g)	Pseudo-second-order model			
		q_e_._cal_ (mg/g)	k min^−1^	Chi^2^	R^2^		q_e_._cal_ (mg/g)	k mgg^−1^ min^−1/2^)	Chi^2^	R^2^
3.47	6.047	8.099	0.187	0.437	0.93	6.047	9.218	0.027	0.209	0.96
11.10	14.88	17.538	0.126	0.517	0.98	14.88	20.61	0.007	0.104	0.99
11.64	17.83	18.836	0.064	0.379	0.99	17.836	23.42	0.002	0.398	0.99

#### Pseudo-first-order model

**Figure 11:**
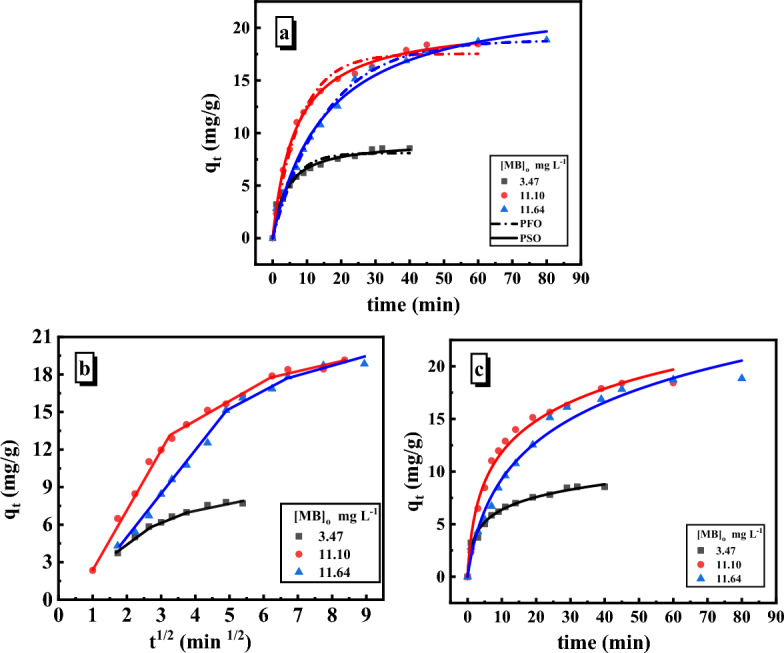
Nonlinear adsorption kinetic plots of MB adsorption on SiO_2_/PANI-SDS nanocomposite. (**a**) Pseudo-First-order kinetic (PFO), Pseudo-second-order kinetic (PSO). (**b**) linear intra-particle-diffusion model. (**c**) Nonlinear Elovich model. Conditions are: [MB]_o_ = 6.36 mg/L, dose = 0.02 g, pH 6, T = 30 °C.

The pseudo-first-order non-linear form of the model is expressed as

9 $${\mathrm{q}}_{\mathrm{t}}={\mathrm{q}}_{\mathrm{e }}\left(1-{\mathrm{e}}^{-{\mathrm{k}}_{1}\mathrm{t}}\right),$$

where q_t_ (mg/g) and q_e_ (mg/g) are the adsorption capacities of the SiO_2_/PANI–SDS nanocomposite at time t (min) and equilibrium. k_1_ (min^−1^) is the pseudo-first-order rate constant. To calculate values of k_1_ and q_e,_ q_t_ was plotted against t and illustrated in Fig. 11a. Based on the results, the value of q_e. cal_ has a significant disparity from the relevant value of q_e.exp_. Additionally, most adsorption data have low correlation coefficient (R^2^) values as given in Table 3. Therefore, it was concluded that the MB adsorption process onto SiO_2_/PANI–SDS did not fit well with the pseudo-first-order kinetics^38^.

#### Pseudo-second-order model

The non-linear form of this model is represented by,

10 $${\mathrm{q}}_{\mathrm{t}}= \frac{{{\mathrm{q}}_{\mathrm{e}}}^{2} {\mathrm{k}}_{2}\mathrm{ t}}{1+{\mathrm{q}}_{\mathrm{e }}{\mathrm{k}}_{2}\mathrm{ t}}.$$

k_2_ (g/mg) is the pseudo-second-order rate constant. k_2_ and q_e_ can be obtained by graphing q_t_ against t as depicted in Fig. 11a. The results obtained from all concentrations of MB show high values of the R^2^, and the q_e.cal_ values are very near to the q_e.exp_ values, Table 3. According to these findings, the pseudo-second-order kinetic model is the best model that describes well the MB adsorption on the SiO_2_/PANI-SDS nanocomposite^39^.

#### Intra-particle diffusion model

The intra-particle diffusion model developed by Weber and Morris^40^ was applied to investigate the kinetics of MB adsorption on the SiO_2_/PANI–SDS surface. This model is expressed in the following equation.

11 $${\mathrm{q}}_{\mathrm{t}}={\mathrm{k}}_{\mathrm{p }}{\mathrm{t}}^{0.5}+\mathrm{c },$$

where, c (mol/g) is a constant associated with the thickness of the boundary layer, and k_p_ (mol/g min^0.5^) refers to the intra-particle diffusion rate constant. Values of these constants determined from the slope and intercept of the q_t_ vs. t^0.5^ plot shown in Fig. 11b are provided in Table 4. Based on this model, if a straight line is produced by plotting q_t_ vs. t^0.5^, the intra-particle diffusion is involved in the adsorption process. However, when this line intersects the origin, the intra-particle diffusion is the only rate-limiting step in the adsorption process^41^. Indicating that intra-particle diffusion was not the only rate-limiting step and that the MB adsorption process involved three steps rather than one, the straight segments in the plot do not pass through the origin. The first segment is attributable to the diffusion of MB molecules through the film to the SiO_2_/PANI-SDS exterior surface (film diffusion), and it is characterized by k_p1_. The second segment is due to the diffusion of MB molecules within the SiO_2_/PANI–SDS pores (intra-particle diffusion), measured as k_p2_. The final segment depicts the slow adsorption of MB molecules into the interior particles of the SiO_2_/PANI–SDS. Consequently, the adsorption of MB decreases until it reaches an equilibrium state at the nanocomposite/liquid interface. Furthermore, values of adsorption rates of the three stages showed that the MB adsorption process was originally faster and subsequently slowed down with time^42^.

**Table 4:** Parameters of intraparticle diffusion and Elovich kinetic models for adsorption of MB on SiO_2_/PANI-SDS (0.02 g), pH 6 ± 0.1, T = 30 °C.

[MB]_o_ (mg/L)	Intraparticle diffusion model				Elovich model			
	kp_1_ (mg g^−1^ min^0.5^)	R^2^	kp_2_ (mg g^−1^ min^0.5^)	R^2^	α (mg g^−1^ min^−1^)	β (g mg^−1^)	Chi^2^	R^2^
3.471	2.307	0.99	1.077	0.99	6.336	0.566	0.07	0.98
11.107	6.614	0.99	1.473	0.98	5.684	0.219	0.51	0.98
11.640	2.915	0.99	0.452	0.94	2.223	0.166	0.75	0.98

#### The Elovich model

Elovich’s empirical model applies to a broad range of adsorption systems with heterogeneous adsorbent surfaces and is frequently valid for chemisorption kinetics, such as electron exchange in the liquid phase. This model relies on the adsorption site energy heterogeneity in the rectangular distribution form^43^. The Elovich nonlinear equation is given as,

12 $${\text{q}}_{\text{t}}=\frac{1}{\beta }\mathrm{ln }\left(1 +\mathrm{ \alpha \beta t}\right),$$

where α is the initial adsorption rate, (mg g^−1^ min^−1^), and β is the desorption constant and denotes the surface coverage extent and energy of activation for chemisorption (g mg^−1^). Figure 11c depicts the nonlinear plots of q_t_ *vs*. t. The constants α and β were determined and are presented in Table 4. The model fit well the experimental data, indicating that the MB adsorption was chemical on the SiO_2_/PANI–SDS surface. Since the values of α are greater than those of β, then the adsorption process is feasible and has irreversible feature^44^.

### Adsorption isotherm

Adsorption isotherms explain how the adsorbent interacts with the adsorbate during the equilibrium state of the adsorption process. It also depicts the distribution of adsorbate molecules between liquid and solid. It is critical to fit the experimental data into several isotherm models to characterize the adsorption phenomenon. Thus, the adsorption of MB onto SiO_2_/PANI–SDS was studied using the most commonly used isotherm models, Langmuir, Freundlich, and Dubinin–Radushkevich (D–R)^45^.

#### Langmuir isotherm model

The model illustrates the existence of a homogenous adsorption process and assumes that the adsorbate molecules will adsorb in a monolayer on the adsorbent surface. According to the Langmuir theory, there are a set number of equally energetic sites on the adsorbent surface, the adsorbent molecules do not interact with the other molecules, and each adsorbent site can only store one adsorbate molecule. Hence the adsorption is a monolayer adsorption^46^. The Langmuir equation is,

13 $$\frac{{\mathrm{C}}_{\mathrm{e}}}{{\mathrm{q}}_{\mathrm{e}}}=\frac{1}{{\mathrm{K}}_{\mathrm{L }}{\mathrm{q}}_{\mathrm{m}}}+\frac{{\mathrm{C}}_{\mathrm{e}}}{{\mathrm{q}}_{\mathrm{m}}}.$$

**Figure 12:**
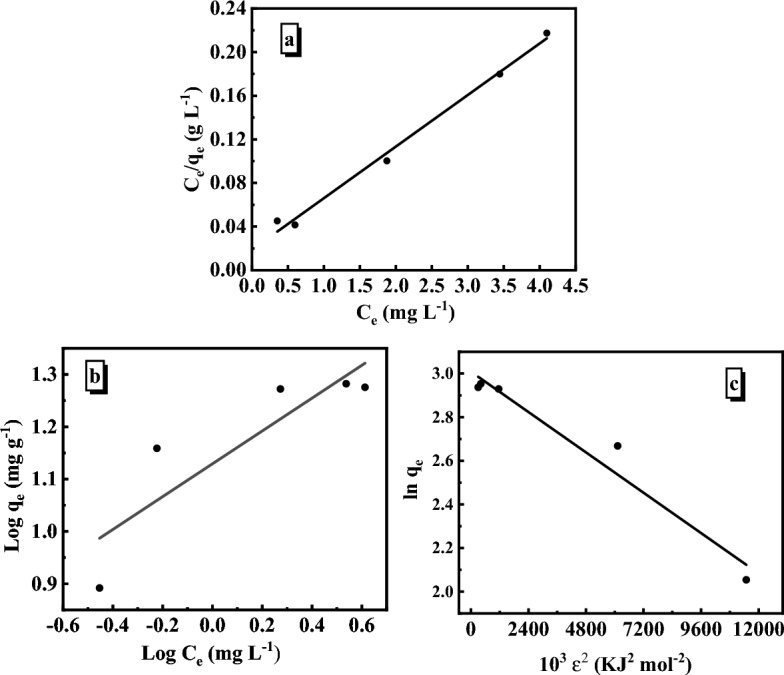
Adsorption isotherm plots of MB on SiO_2_/PANI-SDS nanocomposite. (**a**) Langmuir, (**b**) Freundlich, (**c**) Dubinin-Radushkevich. [MB]_o_ = 6.36 mg/L, dose = 0.02 g, pH 6, T 30 °C.

C_e_ (mg/L) represents the concentration of MB in solution at equilibrium. q_e_ (mg/g) is the adsorption capacity of MB at equilibrium, q_m_ is the maximum adsorption capacity, and K_L_ is the Langmuir constant. Figure 12a shows the plot of C_e_/q_e_ vs. C_e_ from which the values of K_L_ and q_m_ are determined. These values are summarized in Table 5. In addition, the dimensionless factor R_L_ was also calculated to evaluate the feasibility of the MB adsorption on the SiO_2_/PANI-SDS surface. The factor is defined by Eq. (12).

**Table 5:** Adsorption isotherms parameters of MB adsorption on SiO_2_/PANI-SDS, dose = 0.02 g, T = 30 °C.

Isotherm	Parameter	Value
Langmuir	K_L_	4.44 L mg^−1^
	q_m_	24.90 mg g^−1^
	R^2^	0.989
Freundlich	K_F_	18.92 mg g^−1^ (L mg^−1^)^1/n^
	1/n	0.26 L g^−1^
	R^2^	0.953
Dubinin–Radushkevich (D–R)	q_s_	24.90 mg g^−1^
	β	0.0085 mol^2^ kJ^−2^
	E	7.63 kJ mol^−1^
	R^2^	0.970

14 $${\mathrm{R}}_{\mathrm{L}}=\frac{1}{1+{\mathrm{K}}_{\mathrm{L}}{\mathrm{C}}_{\mathrm{e}}}.$$

From the R_L_ value, the features of MB adsorption can be determined. If 0 < R_L_ < 1, the adsorption is favorable, R_L_ > 1 it is unfavorable, linear at R_L_ = 1, and irreversible when R_L_ = 0. Since R_L_ = 0.16 in the present study it then verifies the favorability of MB adsorption on the SiO_2_/PANI-SDS surface.

#### The Freundlich isotherm model

Unlike the Langmuir isotherm model, the Freundlich model describes the adsorption characteristics of the multilayer and heterogeneous surfaces. It is assumed that the strongest active sites are first occupied. Subsequently, the binding strength of the active sites decreases with increasing occupation^47^. Its mathematical expression is given in Eq. (13).

15 $$\mathrm{log }{\mathrm{q}}_{\mathrm{e}}={\mathrm{logK}}_{\mathrm{F}}+\frac{1}{\mathrm{n}} {\mathrm{logC}}_{\mathrm{e}}.$$

K_F_ (mg g^−1^) is the Freundlich constant which refers to the intensity of adsorption while 1/n denotes the favorability of adsorption. Both constants are determined from the slope and intercept of the plot of log C_e_ vs. log q_e_ shown in Fig. 12b. In the case of 0 < 1/n > 1, the adsorption is favorable. When 1/n is zero, the isotherm is irreversible. If 1/n > 1, the isotherm is unfavorable^48^. In this system, the value of 1/n is 0.313, thus indicating that MB has favored adsorption on the surface of the SiO_2_/PANI–SDS (Table 5).

#### The Dubinin–Radushkevich (D–R) isotherm model

The D–R model was developed to deal with the vapor adsorption on the solids with micropores via the pore-filling mechanism. Subsequently, it was developed based on the Polanyi theory and the assumption that the pore distribution in the adsorbent follows the Gaussian energy distribution to describe the adsorption on the heterogeneous surface. Unlike layer-by-layer adsorption on pore walls, the adsorption occurs by filling the micropore volume. Because it distinguishes between physical and chemical adsorption and does not need a homogeneous surface or a constant adsorption potential, the D–R isotherm model works better than the Langmuir and Freundlich models^49^. The following equations provide the linear form of the isotherm.

16 $$\upvarepsilon =\mathrm{RT ln }\left(1+\frac{1}{{\mathrm{C}}_{\mathrm{e}}}\right),$$

17 $${\mathrm{lnq}}_{\mathrm{e}}={\mathrm{lnq}}_{\mathrm{s}}-\upbeta {\upvarepsilon }^{2},$$

where, q_e_ (mg/g) is the amount of MB adsorbed at equilibrium, ε (kJ/mol) is the Polanyi sorption potential, and q_s_ (mg/g) is the theoretical saturation capacity of the SiO_2_/PANI–SDS nanocomposite. The constant β is related to the adsorption energy (E) as given in Eq. (18). The slope and intercept of the ln q_e_ vs. ε plot were used to determine the q_m_ value (Fig. 12c). In contrast the mean free energy (E) of adsorption is given as,

18 $$\mathrm{E}=\frac{1}{({2\upbeta )}^{0.5}}.$$

E (kJ/mol) is the energy required to remove the molecules from the SiO_2_/PANI–SDS surface. Therefore, if E < 8, the adsorption is physical and it is chemical adsorption when 8 < E < 16 kJ/mol^50^. Since the estimated value of E = 7.63 kJ/mol (Table 5), demonstrates that MB was physically adsorbed on the surface of SiO_2_/PANI-SDS.

### Reusability of the nanocomposite

**Figure 13:**
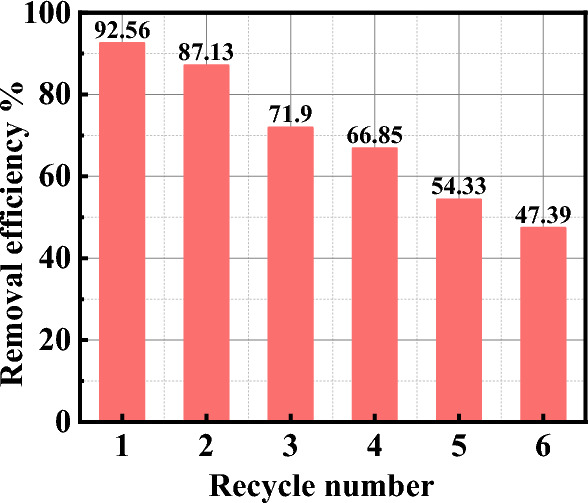
Recyclability of SiO_2_/PANI-SDS nanocomposite for the removal of MB from solution. [MB]_o_ = 6.36 mg/L, dose = 0.02 g, pH 6, at 30 °C.

The regeneration process is crucial in developing economical and cost-effective adsorbents. The results revealed that the SiO_2_/PANI–SDS nanocomposite has low adsorption capacity at low pH. Therefore, acid treatment might be an appropriate approach for the nanocomposite regeneration. Figure 13 depicts the adsorption efficiency of the SiO_2_/PANI–SDS nanocomposite throughout six successive cycles of the MB adsorption–desorption process. The results reveal a depletion in the adsorption efficiency of the nanocomposite across the cycles. Its activity decreased from ~ 92 to 71% along the first three cycles and from ~ 66 to 47% over the next three cycles. Taking into account this behavior of the SiO_2_/PANI–SDS/MB system it can be concluded that such nanocomposite still has a potential for large-scale application in the depollution of wastewater with low costs^51^.

### Adsorption mechanism

**Figure 14:**
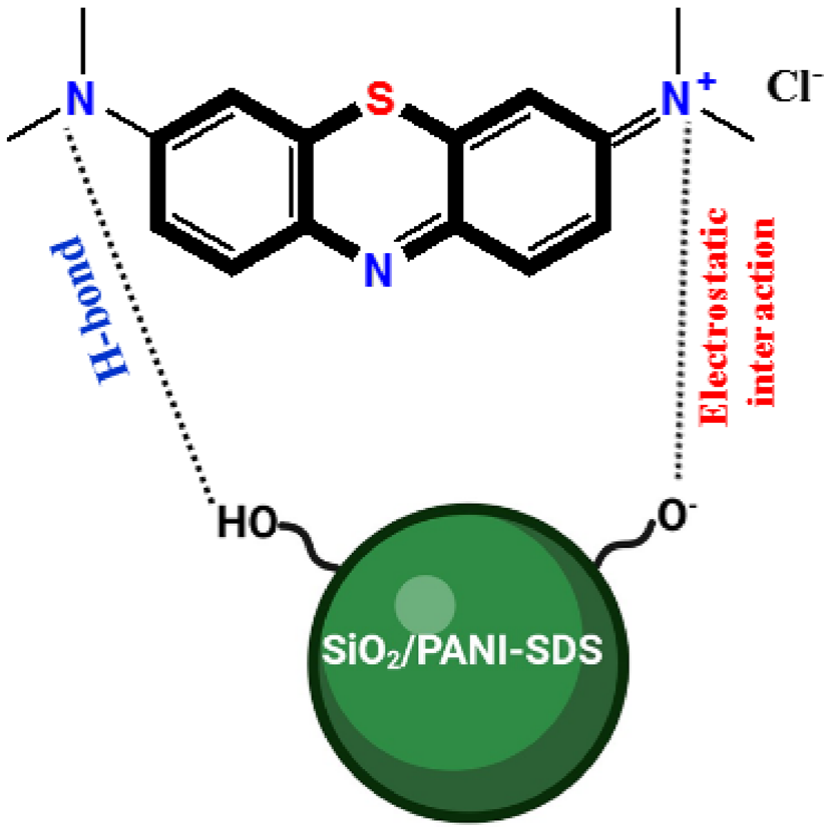
Proposed mechanism for adsorption of MB on SiO_2_/PANI-SDS nanocomposite.

To predict how the MB molecules, get adsorbed on the surface of the SiO_2_/PANI–SDS nanocomposite, it is important to identify the active functional groups on the nanocomposite surface that can interact with the MB molecules. These active functional groups were identified by the FT-IR and XPS analysis. From the FT-IR results, the nanocomposite has S=O, Si–O^–^, and –NH^+^–, demonstrating a high degree of electron delocalization on the SiO_2_/PANI–SDS and a strong interchain NH^+^–N– hydrogen bonding. The XPS data of the deconvoluted peaks of Si, S and O atoms also confirm the existence of Si–O, S–O, H–O, and Si–NO functional groups that are associated with the nanocomposite surface. Therefore, the adsorption of MB by the nanocomposite may proceed via hydrogen bonding and electrostatic interaction. The electrostatic interaction is formed between the Si–O, Si–NO, and S–O groups of the nanocomposite, and the –N^+^– of the dimethylamino group in the MB^52^. The intermolecular hydrogen bond in the form of –N…H–O– forms between the –N– of dimethylamino group of the MB and the Si–O, Si–NO, and S–O on the nanocomposite. At pH 3, the nanocomposite exhibits very low removal efficiency relative to that observed at higher pH, (Fig. 8a). At low pH, the ionized H^+^ ions in the reaction medium tend to bind via an electrostatic interaction with the Si–O, Si–NO, and S–O moieties of the nanocomposites surface faster than the cationic MB^+^ leading to a decrease in the removal efficiency. Above pH 3, the removal efficiency increases with increasing the pH, suggesting that the Si–OH silanol groups becomes more deprotonated to generate the Si–O^–^. The Si–O^–^ groups increase more and more in the reaction medium in addition to the Si–NO and S–O groups of the nanocomposite^53^. These groups possess strong electrostatic interactions with the –N^+^– centres of MB molecules. Hence the removal efficiency is enhanced. A proposed mechanism for the MB adsorption onto the SiO_2_/PANI–SDS nanocomposite is illustrated in Fig. 14.

## Conclusion

The current study demonstrated the successful synthesis of a novel SiO_2_/PANI-SDS nanocomposite and assessed its efficiency for MB removal by batch mode experiment. At pH 6, 6.36 mg/L of MB, and 2.5 g/L of SiO_2_/PANI-SDS dose, the SiO_2_/PANI-SDS nanocomposite can remove ~ 92.5% of the MB within 60 min. The initial MB concentration, nanocomposite dose, pH of the solution, presence of NaCl, and temperature had a great influence on the adsorption process. The removal percentage increased with increasing the dose, pH, and temperature while decreasing with increasing the dye and NaCl concentrations. The kinetic study indicated that the MB adsorption follows the pseudo-second-order and Elovich models. The adsorption process was spontaneous, favorable, and endothermic. It followed Langmuir isotherm with a good fit and maximum adsorption capacity of 24.9 mg/g. According to the Dubinin–Radushkevich isotherm, the micro pores of the SiO_2_/PANI-SDS were first filled with the MB molecules instead of the layer-by-layer adsorption. After four adsorption/desorption cycles, the efficiency of the generated SiO_2_/PANI-SDS nanocomposite only decreased by about 26%. Because of these outstanding properties of the SiO_2_/PANI-SDS nanocomposite, it can thus be considered a cost-effective adsorbent for wastewater treatment.

### Consent to participate

All the authors are agreed to participate in this work.

## Data Availability

All the data and materials are available in the manuscript.

## References

[1] Abdullah T (2021). Preparation and characterization of MnO_2_-based nanoparticles at different annealing temperatures and their application in dye removal from water. Int. J. Environ. Sci. Technol..

[2] Nasar A, Mashkoor F (2019). Application of polyaniline-based adsorbents for dye removal from water and wastewater—A review. Environ. Sci. Pollut. Res..

[3] Ding J (2020). Synergetic adsorption and electrochemical classified recycling of Cr(VI) and dyes in synthetic dyeing wastewater. J. Chem. Eng..

[4] Siadatnasab F, Karami K, Khataee A (2019). Keggin-type polyoxometalates supported on PANI-coated CuS: Synthesis, characterization and application as the efficient adsorbents for selective dye removal. J. Ind. Eng. Chem..

[5] Wang N (2019). Removal of methylene blue by polyaniline/TiO_2_ hydrate: Adsorption kinetic, isotherm and mechanism studies. Powder Technol..

[6] Yan B (2015). Fabrication of polyaniline hydrogel: Synthesis, characterization and adsorption of methylene blue. Appl. Surf. Sci..

[7] Kumar N, Bahl T, Kumar R (2020). Study of the methylene blue adsorption mechanism using ZrO2/polyaniline nanocomposite. Nano Express.

[8] Ismail A (2021). A facile approach to synthesis of silica nanoparticles from silica sand and their application as superhydrophobic material. J. Asian Ceram. Soc..

[9] Farrukh A (2014). Surface-functionalized silica gel adsorbents for efficient remediation of cationic dyes. Pure Appl. Chem..

[10] El-Sawy AM (2021). Catalytic degradation of methylene blue in aqueous solution by H_2_O_2_ and SiO_2_-NH_2_-Cu (II)@ SiO_2_ nanoparticles as catalyst. J. Mol. Liq..

[11] Ingrachen-Brahmi D, Belkacemi H, Mahtout AJ (2020). Adsorption of methylene blue on silica gel derived from Algerian siliceous by-product of kaolin. J. Mater. Environ. Sci..

[12] Liu S (2020). Low-cost route for preparing carbon–silica composite mesoporous material from coal gasification slag: Synthesis, characterization and application in purifying dye wastewater. Arab. J. Sci. Eng..

[13] Kumar A (2020). Synthesis and thermal analysis of polyaniline (PANI). J. Phys. Conf. Ser..

[14] Sandaruwan C (2018). Polyaniline/palladium nanohybrids for moisture and hydrogen detection. Chem. Cent. J..

[15] Noby H (2018). Novel preparation of self-assembled HCl-doped polyaniline nanotubes using compressed CO_2_-assisted polymerization. Polym. J..

[16] Zhu H, Peng S, Jiang W (2013). Electrochemical properties of PANI as single electrode of electrochemical capacitors in acid electrolytes. Sci. World J..

[17] Ashraf SS, Frounchi M, Dadbin S (2020). Gamma irradiated electro-conductive polylactic acid/polyaniline nanofibers. Synth. Met..

[18] He L (2011). Synthesis and characterization of a novel electroactive polymer with oligoaniline and nitrile groups. J. Polym. Res..

[19] Bounedjar M, Naar N, Mekki A (2021). Semi-crystalline polyaniline with an enhanced conductivity synthesized with a novel binary dopant sulfonic acid-surfactant: Mechanical, electrical and shielding performances of Nylon/PANI conductive fabrics at 9.45 GHz. J. Macromol. Sci..

[20] Wang Y (2021). Tuning thermoelectric performance of poly(3,4-ethylenedioxythiophene): Poly (styrene sulfonate)/polyaniline composite films by nanostructure evolution of polyaniline. Polym. Test..

[21] Li H (2021). Tailoring the lateral size of two-dimensional silicon nanomaterials to produce highly stable and efficient deep-blue emissive silicene-like quantum dots. J. Mater. Chem. C.

[22] Shen G (2017). Microwave electromagnetic and absorption properties of SiO_2_/C core/shell composites plated with metal cobalt. Appl. Phys. A.

[23] Xu Z, Yu J, Xiao W (2013). Microemulsion-assisted preparation of a mesoporous ferrihydrite/SiO_2_ composite for the efficient removal of formaldehyde from air. Chem. Eur. J..

[24] Brzeska J (2012). The structure of novel polyurethanes containing synthetic poly [(R, S)-3-hydroxybutyrate]. J. Appl. Polym. Sci..

[25] Zhao Z (2018). Surface-modified shortwave-infrared-emitting nanophotonic reporters for gene-therapy applications. ACS Biomater. Sci. Eng..

[26] Gamal A (2022). Facile fabrication of polyaniline/Pbs nanocomposite for high-performance supercapacitor application. J. Nanomater..

[27] Kumar A (2015). Thermal stability and electrical properties of polyaniline synthesized by oxidative polymerization method. Macromol. Symp..

[28] Rahman SU, Röse P (2021). Exploring the functional properties of sodium phytate doped polyaniline nanofibers modified FTO electrodes for high-performance binder free symmetric supercapacitors. Polymers.

[29] Budi S (2018). Preparation of high surface area and high conductivity polyaniline nanoparticles using chemical oxidation polymerization technique. J. Phys. Conf. Ser..

[30] Ruhi G, Bhandari H, Dhawan SK (2014). Designing of corrosion resistant epoxy coatings embedded with polypyrrole/SiO_2_ composite. Prog. Org. Coat..

[31] Ouachtak H (2023). Combined molecular dynamics simulations and experimental studies of the removal of cationic dyes on the eco-friendly adsorbent of activated carbon decorated montmorillonite Mt@ AC. RSC Adv..

[32] Largo F (2023). Design of organically modified sepiolite and its use as adsorbent for hazardous Malachite Green dye removal from water. Water Air Soil Pollut..

[33] Lei C (2021). Mussel-inspired synthesis of magnetic carboxymethyl chitosan aerogel for removal cationic and anionic dyes from aqueous solution. Polym. J..

[34] Ihsanullah I, Bilal M, Jamal A (2022). Recent developments in the removal of dyes from water by starch-based adsorbents. Chem. Rec..

[35] Mary Ealias A, Saravanakumar M (2019). A critical review on ultrasonic-assisted dye adsorption: Mass transfer, half-life and half-capacity concentration approach with future industrial perspectives. Crit. Rev. Environ. Sci. Technol..

[36] Karmaker S, Nag AJ, Saha TK (2020). Adsorption of reactive blue 4 dye onto chitosan 10B in aqueous solution: Kinetic modeling and isotherm analysis. Russ. J. Phys. Chem. A.

[37] Paluri P, Durbha KS (2021). Equilibrium, kinetic, and thermodynamic study for the adsorption of methylene blue onto activated carbons prepared from the banana root through chemical activation with phosphoric acid. Biomass Convers. Biorefin..

[38] Jiang R (2021). Magnetic NiFe_2_O_4_/MWCNTs functionalized cellulose bioadsorbent with enhanced adsorption property and rapid separation. Carbohydr. Polym..

[39] Zhao Y (2021). Enhanced adsorption of Rhodamine B on modified oil-based drill cutting ash: Characterization, adsorption kinetics, and adsorption isotherm. ACS Omega.

[40] Weber WJ, Morris JC (1963). Kinetics of adsorption on carbon from solution. Sanit. Eng. Div..

[41] Senthil Kumar P (2014). Adsorption of basic dye onto raw and surface-modified agricultural waste. Environ. Prog. Sustain..

[42] Liu Q-X (2019). Adsorption of methylene blue from aqueous solution onto viscose-based activated carbon fiber felts: Kinetics and equilibrium studies. Adsorp. Sci. Technol..

[43] Lin D (2020). Adsorption of dye by waste black tea powder: Parameters, kinetic, equilibrium, and thermodynamic studies. J. Chem..

[44] Ma P (2019). Effect of bifunctional acid on the porosity improvement of biomass-derived activated carbon for methylene blue adsorption. Environ. Sci. Pollut. Res..

[45] Unugul T, Nigiz FU (2020). Preparation and characterization an active carbon adsorbent from waste mandarin peel and determination of adsorption behavior on removal of synthetic dye solutions. Water Air Soil Pollut..

[46] Bo L (2021). A novel adsorbent Auricularia Auricular for the removal of methylene blue from aqueous solution: Isotherm and kinetics studies. Environ. Technol. Innov..

[47] Belhaj AF (2021). Experimental investigation, binary modelling and artificial neural network prediction of surfactant adsorption for enhanced oil recovery application. J. Chem. Eng..

[48] Vaid V, Jindal R (2022). An efficient pH-responsive kappa-carrageenan/tamarind kernel powder hydrogel for the removal of brilliant green and rose Bengal from aqueous solution. J. Appl. Polym. Sci..

[49] Hu Q, Zhang Z (2019). Application of Dubinin–Radushkevich isotherm model at the solid/solution interface: A theoretical analysis. J. Mol. Liq..

[50] Nandiyanto ABD (2022). Isotherm adsorption of 40-μm zeolite particles for treatment of dye wastewater. JESTEC.

[51] Pete S, Kattil RA, Thomas L (2021). Polyaniline-multiwalled carbon nanotubes (PANI-MWCNTs) composite revisited: An efficient and reusable material for methyl orange dye removal. Diam. Relat. Mater..

[52] Yuan N (2019). Adsorptive removal of methylene blue from aqueous solution using coal fly ash-derived mesoporous silica material. Adsorp. Sci. Technol..

[53] Mittal H (2020). Low-temperature synthesis of magnetic carbonaceous materials coated with nanosilica for rapid adsorption of methylene blue. ACS Omega.

